# Are Vaccines the Solution for Methane Emissions from Ruminants? A Systematic Review

**DOI:** 10.3390/vaccines8030460

**Published:** 2020-08-20

**Authors:** Victoria Baca-González, Patricia Asensio-Calavia, Sergio González-Acosta, Jose Manuel Pérez de la Lastra, Antonio Morales de la Nuez

**Affiliations:** 1 Biotechnology of Macromolecules Research Group, Instituto de Productos Naturales y Agrobiología (IPNA-CSIC), 38206 San Cristóbal de la Laguna, Spain; victoria@ipna.csic.es (V.B.-G.); sgacosta010@gmail.com (S.G.-A.); jm.perezdelalastra@csic.es (J.M.P.d.l.L.); 2 Biological Activity Service, Instituto de Productos Naturales y Agrobiología (IPNA-CSIC), 38206 San Cristóbal de la Laguna, Spain; patriciaac@ipna.csic.es

**Keywords:** archaea, greenhouse-gas mitigation, rumen, immunization, antimethanogen

## Abstract

Ruminants produce considerable amounts of methane during their digestive process, which makes the livestock industry as one of the largest sources of anthropogenic greenhouse gases. To tackle this situation, several solutions have been proposed, including vaccination of ruminants against microorganisms responsible for methane synthesis in the rumen. In this review, we summarize the research done on this topic and describe the state of the art of this strategy. The different steps implied in this approach are described: experimental design, animal model (species, age), antigen (whole cells, cell parts, recombinant proteins, peptides), adjuvant (Freund’s, Montanide, saponin, among others), vaccination schedule (booster intervals and numbers) and measurements of treatment success (immunoglobulin titers and/or effects on methanogens and methane production). Highlighting both the advances made and knowledge gaps in the use of vaccines to inhibit ruminant methanogen activity, this research review opens the door to future studies. This will enable improvements in the methodology and systemic approaches so as to ensure the success of this proposal for the sustainable mitigation of methane emission.

## 1. Introduction

Methane (CH_4_) is one of the main greenhouse gases; its negative effect on global warming is 21 times greater than that of carbon dioxide (CO_2_) [1]. Moreover, livestock-keeping is the human activity that generates most CH_4_, since ruminants emit large amounts in their digestive processes. This gas is formed in the ruminant forestomach (rumen) by methanogenic archaea [2]. During normal rumen function, plant material is degraded to produce volatile fatty acids, ammonia, hydrogen (H_2_), and CO_2_. Rumen methanogens principally consume H_2_ to reduce CO_2_ to CH_4_ [3]. Cattle, buffalo, and small ruminants release the equivalent of 2448 million tons of CO_2_ from both enteric processes and manure fermentation [4]. Within the farm environment, enteric fermentation is the most important source of CH_4_ emissions [5]. Thus, enteric CH_4_ generated in the gastrointestinal tracts of livestock is the single largest source of anthropogenic CH_4_ [6]. In the rumen, numerous prokaryotic (bacteria and archaea) and eukaryotic microorganisms (protozoa and fungi) work together to degrade the feedstuff consumed by the host ruminant [7]. In fact, on a well-managed confinement farm, enteric fermentation contributes about 45% of the total emission of greenhouse gases by the whole system. On more extensive grazing farms, these greenhouse-gas emissions could be even higher. For example, increased milk production has a positive correlation with CH_4_ emission [8]. Given that the livestock sector is one of the fastest-growing parts of the worldwide agricultural economy [9], the demand for milk and dairy products is expected to increase in coming decades, and thus so too are the CH_4_ emissions. It is therefore of utmost importance to find ways to mitigate the CH_4_ emissions from enteric fermentation. Mitigation approaches targeted at reducing CH_4_ must consider their effects on both enteric and manure fermentation, which account for approximately 90% and 10% of CH_4_ emissions, respectively [6]. Common approaches to reduce CH_4_ emissions in ruminants include dietary manipulation, drugs to reduce or control the quantity of methanogenic microorganisms in the gut, and/or vaccination. However, current strategies to inhibit methanogen activities in the rumen typically fail or have limited success due to low efficacy, poor selectivity, microorganism resistance, toxicity, or side effects of the compounds or drugs in the host species [3]. Dietary modification is the most-used strategy to reduce CH_4_ in ruminants, taking into account that different concentrates, subproducts, and/or forage combinations can reduce the quantity of CH_4_ production from the rumen [10,11,12], e.g., Goetsch [13] theorized that plant secondary metabolites could decrease CH_4_ emission, permitting the use of H_2_ to increase propionate production.

The control of animal diseases utilizes several strategies. Vaccines are one of the most important approaches, particularly on livestock farms [14]. The use of vaccines in these production sectors is increasing every year, especially for zoonotic diseases and those with significant effects on international trade [15]. However, concern regarding climate change has also increased dramatically. Reduction of emissions could therefore become economically attractive in the near future, making it viable to produce and market vaccines to mitigate climate change. This review attempts to clarify the state of the art of vaccination as a possible method for CH_4_ mitigation in ruminants.

## 2. The Rumen Microbiota 

The rumen functions as a “fermentation chamber”, maintaining the right environment to host a wide community of microbes able to digest lignocellulosic polymers, the main constituent of the ruminant diet. The diet defines the microbial balance in the rumen, and consequently CH_4_ production [16]. An anaerobic atmosphere is maintained, with constant temperature and acidity [17]. Under these conditions, diverse microbes thrive and complex relationships are built between them, including symbiosis, consortia, cross-feeding, etc. [18,19,20]. Together they are able to process plant polysaccharides, which are otherwise indigestible for ruminants [21,22]. These polymers are broken down into products that will serve as nutrients for the animal, such as the volatile fatty acids acetate, propionate, and butyrate [23]. The rumen microbiota also serves other functions like detoxifying substances such as urea and protecting the host from harmful organisms like parasites and pathogens [24,25]. On the other hand, due to their fermentation activity, they generate byproducts such as CO_2_ or the gas of our concern: CH_4_ [26].

The main source of CH_4_ in the rumen is the hydrogenotrophic pathway [27], which is briefly explained as follows. During rumen fermentation, H_2_ is released by various microorganisms from the reducing equivalents in the process of glycolysis and pyruvate oxidative decarboxylation to acetyl CoA. The dissolved H_2_ is transferred between microorganisms in the rumen [28] and can be used by particular microbes in a number of ways, including the reduction of compounds such as fumarate, sulfate, nitrate, or nitrite, or other biochemical reactions such as reductive acetogenesis or hydrogenation of unsaturated fatty acids. However, the main H_2_ sink is CH_4_ generation by methanogens [29,30] in a chemical reaction involving CO_2_ [31]. A higher amount of dissolved H_2_ in the rumen means an increase in CH_4_ production [32], and inhibition of methanogen activity is linked to a decrease in CH_4_ production and an increase in the amount of H_2_ [33]. In addition to the hydrogenotrophic pathway, other metabolic routes for CH_4_ production in the rumen have been described. Some methanogens use the formate remaining from the acetyl-CoA pathway, and, much less commonly, CH_4_ is produced via the methylotrophic pathway (from methyl groups and a certain amount of H_2_) and the acetoclastic pathway (using acetate) [31,34].

It has been suggested that changes in the composition of the microbial communities hosted in the rumen are associated with alterations in CH_4_ production [35]. To understand the process of CH_4_ production, it is necessary to gain insight into this community, which comprises a variety of anaerobic organisms including bacteria, archaea, protozoa, anaerobic fungi, mycoplasmas, and viruses [36,37]. Newborn ruminants have no rumen microorganisms at birth, but they acquire them in their first days of life, during the lactation period [38,39]. First, bacteria and archaea are established in the rumen, even before ingestion of solid foods [40]. Shortly afterwards, anaerobic fungi appear, and finally ciliate protozoa, the group that takes longest to stabilize even after weaning [41]. After the microbiome is established, it is thought to remain stable throughout the life of the ruminant [42,43], although recent studies have challenged this [44]. There is controversy regarding the factors that affect this microbiota; many have been mentioned in the literature, including diet, animal age, antibiotics, animal health, location, season, and host [37,41,45].

The most abundant microbes in biomass terms are bacteria, which are also highly diverse [41]. Their most common phyla are Firmicutes, Bacteroidetes, and Proteobacteria [46]. Although bacteria in the rumen are not direct CH_4_ producers, differences in bacterial community structure are associated with these gas emissions. Lower CH_4_ production is associated with higher numbers of species that produce propionate (*Quinella ovalis*), lactate, and succinate (*Fibrobacter* spp.) [47], and higher amounts of certain genera of Proteobacteria phylum [46]. On the other hand, higher methane production is associated with greater numbers of species that are known to produce H_2_ in large amounts, e.g., *Ruminococcus*, *Ruminococcaceae*, *Lachnospiraceae*, *Catabacteriaceae*, *Coprococcus* and other *Clostridiales*, *Prevotella*, and other *Bacteroidales* and *Alphaproteobacteria* [47].

Archaea represent about 0.3 to 3% of the rumen microbiome, and they are also less diverse, with 10 main taxa [48,49,50]. Most (92.3%) are methanogenic, and are responsible for all CH_4_ production in the rumen [51]. Most methanogens belong to four orders: Methanobacteriales, Methanomicrobiales, Methanosarcinales, and one uncultured group called either Rumen cluster C (RCC), Thermoplasmatales-affiliated lineage C (TALC), or Methanoplasmatales [49,52]. The order Methanobacteriales is the most common in the rumen and comprises three major genera: *Methanobrevibacter* (which makes up 60% of the methanogens detected in the rumen [53], *Methanobacterium,* and *Methanosphaera* [18]. The first two are mainly hydrogenotrophic, although they can also use formate to produce CH_4_ [51], and *Methanosphaera* species are methylotrophs [54]. Concerning the other orders, Methanomicrobiales is represented mainly by the genus *Methanomicrobium*, which is found relatively abundantly in the rumen. The most common species belonging to this genus (*M. mobile*) is hydrogenotrophic [29]. The main member of the order Methanosarcinales is the genus *Methanosarcina*, which is methylotrophic and much less abundant than the aforementioned species [52]. The last order, the RCC, is barely known but could be methylotrophic as well [55]. Methanogens can be present in the rumen as free-living microbes, or associated with protozoa (10–20% [56]), either on their surface or endosymbiotically [46]. This portion is thought to produce from 9 to almost 40% of the CH_4_ originating in the rumen [57,58] and these microbes belong mostly to the hydrogenotrophic family Methanobacteriaceae [18].

Up to 12 genera of ciliate protozoa constitute an important part of the rumen microbiota, just behind bacteria in terms of biomass [37,46]. As stated before, there is a close relationship between methanogenic archaea and some protozoa [57], such as *Entodinium*, which is the dominant genus of protozoa in the rumen [59]. Protozoa favor archaeal populations, as they produce large amounts of H_2_ and provide physically protected support for methanogens [20]. However, the role of protozoa in the rumen is unclear. Their absence is associated with an outflow of microbial protein from the rumen, a drift in number and diversity in methanogen populations, and a decrease in CH_4_ production [39,60].

The last group worthy of mention are the anaerobic fungi, represented by nine genera [61], which may contribute up to 10% of the total rumen biomass [62]. Fungi produce H_2_, among other metabolic products [63], and fungi–archaea associations have been reported [61,64]. Despite this, the relationship between fungal abundance and CH_4_ production is not clear [46].

## 3. Antimethanogen Vaccines to Reduce CH_4_ in Ruminants

**Figure 1:**
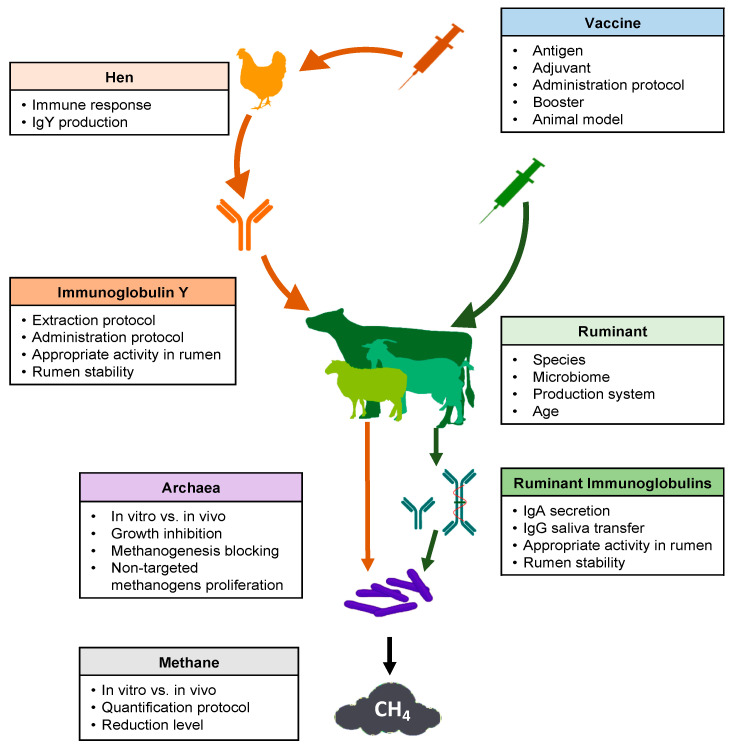
Schematic overview of key points to consider for the use of vaccines to decrease methane emissions from ruminal fermentation.

Several key points should be considered in the development of a successful strategy regarding the use of vaccines to reduce methane production from ruminal fermentation (Figure 1). Many articles and reviews have cited this possibility [26,30,65]. However, experimental research carried out between 1995 and 2020 was scarce in the consulted database (Table 1).

Several problems arose when comparing studies to assess the possibilities of using vaccines for this purpose. Concerning experimental design, as expected, the chosen antigens have developed along with the new technologies in the last 25 years, from whole methanogen cells to recombinant proteins from specific enzymes involved in CH_4_ production. Additionally, the different adjuvants and vaccination protocols used (Table 1) made it difficult to compare results. For example, Wedlock et al. [53] and Subharat et al. [66] both utilized recombinant glycosyl transferase protein (rGT2) as antigen, but the former with saponins as adjuvant and an intramuscular administration route in sheep as experimental animals, while the second was subcutaneous using Montanide in 5 month old calves. Additionally, those studies evaluated different immunoglobulins (IgG, IgA, and IgY) and samples (blood, saliva, and rumen), or analyzed the effect on CH_4_ production using different approaches (in vitro, in vivo).

The most frequently used experimental animal model was the sheep, which was used in 8 out of 11 studies. One of the remaining studies used cattle and another used goats. Finally, a study proposed passive immunization producing antimethanogen Igs in hens. This made it difficult to compare research in order to draw solid conclusions. Patil et al. [67] assayed the immune response of sheep, cattle, and goats against four different serotypes of Foot and mouth disease virus at different times postvaccination. The cows showed higher levels of neutralizing antibodies than small ruminants for all tested virus serotypes. Lobato et al. [68] compared vaccination with recombinant toxin of *Clostridium perfringens* in the three common livestock ruminant species. In this study, sheep showed the highest antibody level, cattle the lowest, and goats intermediate. Moreira et al. [69] tested three recombinant vaccines against alpha, beta, and epsilon toxins of *C. perfringens* in the same three species. They found an interaction between antigens and species. There were no differences between species, except for with epsilon toxin. In the latter, cattle showed the highest antitoxin levels, with no differences between sheep and goats. In the same way, each species had a different response to each recombinant toxin, whereby all these animals had higher values against beta and lower against alpha toxin. Iqbal et al. [70] observed that ruminal bacterial, methanogen, and protozoal communities were different between cattle and buffalo, although *Methanobrevibacter* was the major genus for both species. These studies show that the animal model selected has an interaction with the antigen used. Obviously, small ruminants are cheaper animal models than cattle, and have fast growth and immune maturity. For these reasons, the use of goats and sheep in the early stages of vaccine development is more practical. However, the novel antigen must also be tested in the species for which it is being developed.

Additionally, animal age was another source of variation, with vaccinated sheep ranging from 3–5 months to 5 years old. It is well known that lambs are more susceptible to infectious diseases than adult sheep, and their immune resistance progressively increases during the first year of life [71]. According to Nguyen et al. [72], who compared 3 months old lambs with 2–5 years old sheep following a single intravenous injection of chicken erythrocytes, the adults had higher antibody titers than the young animals. This author affirmed that the antibody response of lambs reached the adult level at age 7–8 months and sex was not a variable that influenced this humoral response. Similarly, Watson et al. [71] assayed the antibody production of weaners and adult sheep against *Brucella abortus*. They reported that adults always showed a higher level of antibodies than weaners. Additionally, those authors found that both CD4+ and CD8+ in lymph and blood were higher in adults than in weaners, but B cells are lower in adult than in weaners’ lymph, with no difference in blood between ages. The authors suggested that B cells are not completely functional in younger animals, leading to the lower antibody response. Shu et al. [73] worked on a vaccine against *Streptococcus bovis* plus Freund’s adjuvant, reporting a lower antibody concentration than the previous studies in sheep. They tentatively attributed this difference to the age of the animals: 6 months old for Gill et al. [74], 1 year old for Shu et al. [75], and 2 years old in Shu et al. [73], where older animals showed higher antibody levels. However, methanogen vaccines in young animals are a very interesting target, because early programming of rumen microbiota using vaccines could be a better solution in comparison to adult animal vaccines. The rumen microbiota is established early in ruminant life, and it is possible to mold it through diet around weaning time, with a long-lasting effect [76]. De Barbieri et al. [77] found that rumen bacterial communities can change in both mothers and lambs after oral rumen inoculation in the neonatal period or first weeks of life.

The choice of the antigen to be inoculated is a key aspect for the development of a vaccine against methanogenic archaea in the rumen. Different approaches have been used to target methanogens (Table 1). The first strategy was to vaccinate the animals with whole cells of different archaeal species found in the rumen. In some studies, they specified that the methanogens had previously been killed by formaldehyde [78,79,80,81] or freeze-dried [82]. Baker and Perth [78] used a mix of ten strains of *Methanobrevibacter ruminantium*, *M. arboriphilus*, *M. smithii*, *Methanobacter formicium*, and *Methanosarcina barkeri.* Wright [79] checked 16S rDNA clone libraries from Australian sheep rumen samples. Based on that information, they chose one vaccine design with three strains of *Methanobrevibacter* spp. (two of them isolated in their lab in Australia) and another vaccine with seven strains from the four *Methanobrevibacter* species, *Methanomicrobium mobile*, *M. barkeri*, and *Methanobacterium formicicum*. Despite promising results by Wright [79], Clark et al. [80] tried to replicate them using the same mixture of three methanogens, alongside a combination of this mix with methanogenic material isolated from New Zealand sheep. Williams et al. [81] used whole cells of three *Methanobrevibacter* strains, *Methanomicrobium mobile*, and *Methanosphaera stadtmaniae*, which altogether comprised more than half of all the methanogen strains detected. Cook et al. [82] used *Methanobrevibacter ruminantium*, *M. smithii*, and *Methanosphaera stadtmaniae*, each in an independent hen group. They compared the in vitro effect of semipurified IgY and freeze-dried egg yolk from hens vaccinated with each archaeal species and a combination of the three.

Another strategy, derived from the first, was to use cell components as antigens. Wedlock et al. [83] compared the use of whole cells with cytoplasmic and wall-fraction proteins from *M. ruminantium*. In parallel, Leahy et al. [84] published the genome sequence of *M. ruminantium*; based on this sequence, these researchers chose nine peptides from extracellular regions of the cited archaea. Those peptides were synthesized and joined to keyhole limpet hemocyanin (KHL), to be used as antigens. Later, Wedlock et al. [53] compared cytoplasmic and wall-fraction proteins with seven peptides from the extracellular domain of SecE and rGT2. The latter protein was used by Subharat et al. [66] and Subharat et al. [85] to vaccinate cattle and sheep. Zhang et al. [86] used the protein EhaF from *M. ruminantium* M1, which was one of the potential antigen candidates identified by Leahy et al. [84], with a key function in hydrogenotrophic methanogenesis.

Obviously, appropriate adjuvants must be selected for successful vaccine performance. This choice is based mainly on the animal species and antigen used. The experiments compiled in this review show how adjuvant use has developed over time, as new experience is acquired. Four out of ten ruminant experiments and the one with hens added complete/incomplete Freund’s adjuvant (FCA/FIA). Another two used saponins, and two recent studies used Montanide ISA. Shu et al. [73] compared the immune response to *S. bovis* vaccine with six different adjuvants (FCA, FIA, QuilA, dextran sulphate, alum, Gerbu). They found that FCA produced the largest quantity of blood antibodies in sheep. Using antimethanogen vaccines, two studies compared the efficacy of different adjuvants. Subharat et al. [85] contrasted four adjuvants (saponin, chitosan, lipid nanoparticles, and Montanide ISA). They reported that Montanide ISA61 produced the most IgG and IgA in saliva and serum. Subharat et al. [66] had previously affirmed that this Montanide with and without monophosphoryl lipid A was able to induce a strong humoral response in both IgA and IgG. The most usual administration route was subcutaneous in ruminants (six out of eleven); intramuscular and intradermal were the next most frequently applied in ruminants (both used in two experiments), and Baker and Perth [78] used intraperitoneal. The route in hens was intramuscular in the hen breast. Intramuscular and subcutaneous administration routes were the most common, although it has been suggested that intradermal injection could improve the mucosal response [87]. This is of great interest concerning the present topic. More research is necessary about the antigen–adjuvant–administration route combinations able to achieve a better combined response.

Regarding the booster and booster time, a significant variation in both number and period is shown in Table 1. Of the vaccination schedules, the most frequently used was one booster (six out of twelve studies) between 21 and 42 days postprimary, followed by two boosters (three out of twelve). The second vaccination given by Wright et al. [79] was not considered a booster because those authors decided to administer it when they observed low antibody levels, and neither was the third vaccination by Subharat et al. [85], since they tested only one group of animals to determine antibody longevity and the effect of boosting. Examining the results, administration of only one or two boosters appears insufficient to provide long-term immunity. For example, Williams et al. [81] reported that one booster 28 days after primary provided a peak at Day 55 after primary, but the titer decreased by Day 99. Using two boosters, Subharat et al. [85] achieved similar results, with a peak at Day 42 after the primary and the titer decreased until Day 133, when the animals were revaccinated and their specific antibodies titers increased. Those results indicate that a booster is necessary to reinforce antibody secretion. None of the other available studies elucidated the issue in this sense, despite this being a very important piece of knowledge to support this procedure for CH_4_ mitigation.

The time of sample collection to evaluate the immune response was another source of variation. Some authors decided to take only one sample after vaccination to quantify the specific antibodies [83,86], and this did not permit assessment of the specific antibodies’ secretion curves. Therefore, it is not possible to elucidate whether the curves were in their increasing, peak, or decreasing phases. In other studies, which measured immunoglobulins (Igs), the sampling time allowed analysis of the curve and also of the different phases of the antibody curves. Lobato et al. [68] tested a toxin vaccine on sheep, goats, and cattle with a booster on Day 28 after the primary. They reported that no antitoxin antibodies were detected on Day 0. On Day 42, 40% of goats, 60% of sheep, and 80% of cattle had titers lower than 1 IU/mL. On Day 56, all animals had titers equal to or higher than 5.8 IU/mL; sheep had the highest values, followed by goats and cattle.

## 4. Immunoglobulin Production, Saliva Secretion, and Activity in Rumen

In general, the immune response in the mucosa is mediated by mucosal-associated lymphoid tissue. However, no organized lymphoid tissue can be found in the rumen epithelium, and saliva has been suggested to be the main vehicle for introducing Igs into the rumen [40]. The efficacy of vaccine strategies to decrease CH_4_ production in the rumen depends on salivary Ig binding to the methanogen surface epitopes, which must inactivate, impede, or hinder CH_4_ production in the rumen [88]. Around 70% of the water contained in the rumen comes from saliva, which is the major source of antibodies in the rumen contents [74]. Previous authors affirmed that antibodies in serum are an important source of these immune proteins. After the stimulation of antibody production by vaccine, the Ig secretion (mainly IgA and IgG) in saliva is the second bottleneck in mitigation of CH_4_ through vaccination, due to limited IgG transfer from blood to saliva.

Table 1 and Table 2 show that eight of twelve trials measured Igs. All eight measured IgG in blood, seven in saliva, and five in rumen liquor. Only three, three, and one analyzed the mucosal secretory IgA in blood, saliva and rumen liquor, respectively. All trials achieved specific Ig production with different protocols, antigens, and adjuvants. These studies were difficult to compare, because most expressed antibody results as titers against the antigens used, but only a few of them offered results in absolute values as mg/mL. Wright et al. [79] reported the highest levels of antibodies before re-vaccinating animals 153 days after the primary vaccine. Other researchers achieved higher Ig levels with one booster (21 or 28 days after primary) or two (21 and 42 days after primary). The peaks in IgG and IgA were at similar times and the results showed the most IgG in blood, but IgA was higher in the saliva and rumen. When Leahy et al. [84] tested nine vaccines with peptides of *M. ruminantium M1*, they reported all peptides to be antigenic. It is noteworthy that the sheep attained the maximum antibody titers at different times, depending on the peptides. These were four out of nine on Day 42, with two boosters at 14 and 28 days after primary; then another four on Day 84, with four boosters 14, 28, 56 and 70 days after the primary. Finally, one group of animals reached the maximum on Day 98 after receiving five boosters on Days 14, 28, 56, 70, and 84 after the primary. Thus, these data show that different antigens can cause immune reactions at different times, depending on several factors.

The substantial and continuous transfer or production of salivary antibodies will be crucial for the success of an antimethanogen vaccination strategy [66]. Assuming saliva is the principal source of ruminal antibodies, IgG transfer from blood and salivary IgA production are the main objectives of this approach. Secretory IgA has been shown to recognize 20% of commensal bacteria within the rumens of calves [89]. Fouhse et al. [90] hypothesized that if salivary IgA is a potential mechanism to determine commensal rumen microbiota, IgG may play a similar role. Six of the analyzed studies had between 279- and 800-fold more IgG in blood than in saliva. This points to a limited IgG transfer from blood to saliva. The other limitation of this antimethane approach is the survival of immunoglobulins in the rumen. In four of the studies, it was possible to calculate the IgG concentration ratio between saliva and rumen (3.88, 7.69, 84, and 209, in [65,80,84,85], respectively). This ratio was only possible to determine for IgA in two studies: 11.7 [85] and 26.11 [66]. However, IgA production in saliva is not comparable with IgG blood levels. There were contrasting results in these studies, i.e., Wright et al. [79] reported a higher titer of specific IgG in saliva than IgA, while Subharat et al. [85] found that 35% of total IgG was specific against methanogen protein, versus 42% of IgA. Using a rGT2 protein from *M. ruminantium*, Subharat et al. [66] reported a 17,416 and 30 μg/mL of IgG in blood and saliva, respectively, from vaccinated 5 month old male Holstein–Friesian calves. Similarly, the same group with the same antigen reported 19,931 and 41.7 μg/mL of IgG in blood and saliva, respectively, from vaccinated 6 month old lambs. Subharat et al. [66] commented that IgA is more resistant to rumen fluid than IgG, while both can maintain functionality for around 8 h in the rumen, as Williams et al. [59] also reported. However, the same group [85] described one year later that the IgG and IgA decreased by between 50% after 1.5 h incubation and 80–90% by 4 h. Therefore, antibodies induced by the vaccine maintain their activity in the rumen long enough to interact with antigen targets.

## 5. Vaccines and Rumen Populations/CH_4_ Emission

The rumen wall does not present glandular structures and is highly keratinized [91]; for this reason, it has been suggested that humoral immune responses in this organ are absent [74]. As previously mentioned, there is also no secretion of Igs in the rumen; they reach it through saliva [40]. The Igs play multiple roles, including complement fixing, opsonization, blocking, neutralization, and precipitation [92]. As there are no other components of the immune system in the rumen, such as complement or effector cells, the efficacy of the antibodies relies on their capacity to agglutinate and immobilize microorganisms, or to neutralize some essential structures of the microbes. The possibility of using vaccines to alter the microbial community of the rumen has been explored with different purposes. Gnanasampanthan [93] observed immobilization of rumen ciliates in vitro after adding immunized ewe antibodies. Williams et al. [59] targeted certain species of protozoa and recorded binding of antibodies to protozoa in vitro, and a reduction of their numbers. However, when they carried out in vivo trials, the vaccination had no effects on protozoan populations in the rumen. Shu et al. [94] reported milder symptoms (low ruminal pH and diarrhea) of ruminal acidosis in steer immunized with the principal bacteria responsible (*Streptococcus bovis* and *Lactobacillus* spp.). Sheep vaccinated with *S. bovis* also prevented symptoms of this condition [74,75]. Zhao et al. [95] observed less urease activity in cattle immunized with bacterial rumen urease compared to controls, in both in vivo and in vitro essays.

The ultimate aim of the studies covered in this review is for ruminants to produce less CH_4_. There is a wide array of techniques used to measure CH_4_ emissions by ruminants, differing in costs and suitability for the concrete purpose of study [31]. As shown in Table 3, seven out of eleven studies measured the CH_4_ production (three of them used in vitro and four in vivo techniques). Only three of them examined the effect of the vaccines on ruminal populations (two in vivo and one in vitro). Correspondence between results from in vitro and in vivo trials is questionable, and there are studies that both support and oppose this relation [96]. As an example, Bhatta et al. [97] measured CH_4_ production in goats and found a solid relationship between estimates from in vitro systems and the measures from open respiration chambers (in vivo systems). In contrast, Williams et al. [59] found a discrepancy between results in vitro (successful) and in vivo (unsuccessful) when they immunized sheep against rumen protozoa.

Measuring CH_4_ using in vitro gas-production techniques is cheap, fast, and easy to replicate, because variation between samples is reduced compared to in vivo systems. As it is a simplification of real systems, it is recommended as a first approximation that should then be endorsed through experiments in animals [96]. All the in vitro studies showed some effect on CH_4_ production, despite different approaches to the problem. Baker and Perth [78] reported less CH_4_ emission (*p* < 0.018), when they compared ruminal fluid from the same sheep pre- and postvaccination. They also achieved a reduction in CH_4_ when comparing animals vaccinated with methanogen mix vs. adjuvant–PBS, with data both uncorrected (*p* < 0.018) and corrected for dry matter intake (*p* < 0.06). Cook et al. [82] purified chicken antibodies (IgYs) from three groups of hens immunized against three methanogens. Incubating ruminal fluid with these IgYs did not reduce CH_4_ emissions. However, a decrease in CH_4_ was reported when using total egg powder after 12 h incubation. This effect was stronger when applying a combination of eggs against three methanogens instead of using egg against a single strain. The reduction was no longer appreciable at 24 h of ruminal fluid incubation in any group. It is noteworthy that egg from non vaccinated hens caused a reduction in CH_4_ similarly to egg from immunized hens. So, in this particular experiment it seems that egg components other than IgYs caused a CH_4_ decrease. This can be explained because fatty acids (FAs) can inhibit CH_4_ production through various mechanisms; unsaturated FAs compete via H^+^ with methanogens [98], and long-chain FAs are directly toxic to methanogens [99]. Wedlock et al. [83] achieved an inhibition of CH_4_ production when growing *M. ruminantium* with the treated antisera of sheep vaccinated against whole cells, cytoplasmic fraction, or proteins derived from the cell wall. Additionally, they observed that the antisera were able to agglutinate cells of *M. ruminantium*, as well as to inhibit their growth, compared to pre-immune sera. However, the capacity to agglutinate the archaeal cells was not correlated to this inhibition of growth.

In vivo direct systems, which comprise open and closed respiration chambers, are very accurate, and the latter is widely considered the gold-standard method [100]. Nonetheless, they have some disadvantages: the animals are limited in their movements and feeding behavior, results differ from those gathered using free-range animals, and the infrastructure is expensive. In addition, measurements must be taken over short periods of time no longer than three days, and variations in gas production during that period have been repeatedly recorded [101]. In vivo indirect systems like the SF_6_ tracer are widely used alternative techniques, as they overcome some of the disadvantages of the respiration chambers. For example, the animal maintains its grazing habits and it is more economical [100]. However, Wright [79] did not find a clear relationship between SF_6_ and closed respiration chamber measurements. This reflects an inconsistency that has previously been reported [102] and is considered one of the main problems of this method [96].

Regarding the effect of vaccines on CH_4_ evaluated in vivo, Wright [79] used closed respiration chambers and was recorded a 7.7% (*p* < 0.51) reduction in CH_4_ production intake with a vaccine formulation that contained three strains of methanogens. Clark et al. [80] tested Wright’s three-methanogen vaccines, but found no reduction of CH_4_. Although these studies used the same antigens, several differences between them (animal age and location, booster, CH_4_ measuring technique) prevent comparison and a solid conclusion. Williams et al. [81] and Zhang et al. [86] reported no effects of vaccination on CH_4_ production (in sheep with a methanogen mix, and in goats with recombinant protein, respectively) using open-circuit chambers. Both studied the effects of the vaccines on rumen populations. Williams et al. [81] used real-time PCR to calculate numbers and checked clone library data to calculate diversity, but this group found no significant differences in total numbers of methanogens in the rumen of control and treated sheep. The authors suggested that some targeted methanogens could have been affected by the vaccine, as the diversity and methanogen compositions of the population were different in the different groups of sheep. Zhang et al. [86] did not detect alterations in either number or composition of methanogens. As a last remark, most of them measured CH_4_ emission around one month after vaccination or booster: 28 days [80], 28–42 days [59], and 34–42 days [81], except Zhang et al. [86], who measured it 15–17 days after the second booster (Table 1 and Table 3). This is an important source of variation, among others, which impedes comparison between these studies.

## 6. Conclusions

In summary, the possibility of applying vaccines to mitigate CH_4_ production from enteric fermentation in ruminants has been repeatedly suggested. Nevertheless, it is complicated to evaluate the real effectiveness of this strategy. Few studies have directly assessed the complete approach, i.e., from vaccination to enteric animal CH_4_ emission measurement. Furthermore, the great variety in methods is an obstacle in comparison of results from different studies in an appropriate and repeatable way. However, the strategy has been considered promising by many authors, and more research is needed to reach a rigorous conclusion on its feasibility, practical implementation, and sustainability. Various steps should be considered for future studies, such as antigenic capacity, Igs in saliva (IgG transfer and IgA production), action and stability of Igs in the rumen, and, finally, how to evaluate CH_4_ production.

## Figures and Tables

**Table 1:** Summary of experimental designs used in research into vaccination for mitigating methane in ruminants.

Animal Tested	Antigen	Adjuvant	Administration Via	Booster	References
Sheep Weaner wethers	Mix of 10 methanogens, formaldehyde-killed, whole cells	Complete Freund’s adjuvant	Intraperitoneal	28 days after primary	[78]
Sheep 5 years old	Mix of three methanogens, formaldehyde-killed, whole cells	Montanide ISA50	Subcutaneous	153 days after primary	[79]
	Mix of seven methanogens, formaldehyde-killed, whole cells				
Sheep 9 months old	As Wright [79] Mix of three methanogens	Not specified	Not specified	42 days after primary	[80]
	As Wright [79] Mix of three methanogens plus additional methanogenic material isolated from New Zealand sheep				
Hen 24–25 weeks old	Mix of three methanogens, freeze-dried, whole cells	Primary with complete Freund’s adjuvant Booster with incomplete Freund’s adjuvant	Pectoral muscle	21, 42, 84, and 133 days after primary	[82]
		Montanide ISA70		21 and 42 days after primary	
Sheep 2 years old	Mix of five methanogens, formaldehyde-killed, whole cells	Not specified	Subcutaneous	28 and 103 days after primary	[81]
Sheep 9–11 months old	Whole cells of *Methanobrevibacter ruminantium* M1	Primary with complete Freund’s adjuvant Booster with incomplete Freund’s adjuvant	Subcutaneous	21 days after primary	[83]
	Cytoplasmic fraction of *M. ruminantium* M1				
	Wall fraction of *M. ruminantium* M1				
	Wall fraction of *M. ruminantium* M1 with trypsin				
	Wall-fraction-derived-protein *M. ruminantium* M1				
Sheep 1–3 years old	Nine peptides from *M. ruminantium* M1 extracellular regions of eight proteins	Primary and 14 days booster with complete Freund’s adjuvant Other boosters with incomplete Freund’s adjuvant	Intradermal 10–15 sites	14, 28, 56, 70, 84, 98, and 112 days after primary	[84]
Sheep Age not specified	Cytoplasm-derived proteins from *M. ruminantium M1*	Saponin	Subcutaneous	No booster	[53]
	Wall-derived proteins from *M. ruminantium M1*				
Sheep Age not specified	Large extracellular domain of recombinant GT2 of *M. ruminantium* M1	Saponin	Intramuscular	21 days after primary	
	Seven synthetic peptides from extracellular domain of SecE from *M. ruminantium* M1				
Cattle 5 months old	Large extracellular domain of recombinant GT2 of *M. ruminantium* M1	Montanide ISA61	Subcutaneous	21 days after primary	[66]
		Montanide ISA61 plus monophosphoryl lipid A			
Goat 18 months old	Protein recombinant EhaF from *M. ruminantium* M1	Primary with complete Freund’s adjuvant. Booster with incomplete Freund’s adjuvant	Intradermal Eight sites	35 and 45 days after primary	[86]
Sheep 6 months old	Large extracellular domain of recombinant GT2 from *M. ruminantium* M1	Saponin	Intramuscular	21 days after primary	[85]
		Lipid nanoparticles/cationic liposomes	Subcutaneous		
		Chitosan thermogel			
		Montanide ISA61		21 and 133 days after primary	

rGT2 (recombinant glycosyl transferase protein).

**Table 2:** Summary of immunoglobulin use in research into vaccination for methane mitigation in ruminants.

Immunoglobulin	Time to Peak after Primary	Higher Values			IgG–IgA Ratios ^3^	References
		Titer	Time	Conditions		
Blood IgG	27 days	475,000 ^1^	195 days after primary	Primary vaccination with three methanogen species, then revaccination 153 days later.	Blood–saliva IgG: 279 Blood–saliva IgA: 317 Blood IgG–IgA: 5.16 Saliva IgG–IgA: 5.86	[79]
Blood IgA		92,000 ^1^				
Saliva IgG		1700 ^1^	174 days after primary			
Saliva IgA		290 ^1^				
Rumen IgG	Not specified	Detected	119 days after revaccination			
Blood IgG	55 days	540,000 (unit/mL) ^1^	123 days after primary	Primary vaccination with booster at 28 days and revaccination 103 days later.	Blood–saliva IgG: 617 Blood–rumen IgG: 2,348 Saliva–rumen IgG: 3.88	[81]
Saliva IgG		875 (unit/mL) ^1^				
Rumen IgG		230 (unit/mL) ^1^				
Blood IgG	Only one measurement after vaccination	44,800	35 days after primary	Primary vaccination with booster at 21 days after primary. Group vaccinated with cell-wall-derived proteins	Blood–saliva IgG: 800	[83]
Saliva IgG		56				
Blood IgA		Not specified	Not specified	Not specified		
Saliva IgA		Not specified	Not specified	Not specified		
Blood IgG	mtrE peptide 42 days after primary	102,400	84 days after primary	Primary vaccination with booster at 14, 28, 56, and 70 days after primary. Group vaccinated with mtrD peptide		[84]
	mtrC peptide 84 days after primary					
	mtrD peptide 84 days after primary					
Blood IgG	Not specified	1000-fold more that prevaccinated sample	Not specified	Primary vaccination: one group with cytoplasmatic fraction of *M. ruminantium* M1 and second group with cell-wall-derived proteins from the same microorganisms		[53]
Saliva IgG	Not specified	Not specified	Not specified			
Blood IgG	Only one measure after vaccination	Not specified	77 days after primary	Primary vaccination and booster at 21 days after: one group with extracellular domain of GT2 from *M. ruminantium* M1 and second group with extracellular domain of SecE from the same microorganisms		
Saliva IgG						
Rumen IgG						
Blood IgG	21 days after primary	6.5 (log10 units/mL) ^1^	21 days after primary	Primary vaccination with booster at 21 days after primary. One group vaccinated Montanide ISA61 and other group with the same adjuvant plus MPL^2^	Blood–saliva total IgG: 581 Blood–rumen total IgG: 4465 Saliva–rumen total IgG: 7.69 Blood–saliva total IgA: 0.35 Blood–rumen total IgA: 9.36 Saliva–rumen total IgA: 26.1 Blood total IgG–IgA: 97.8 Saliva total IgG–IgA: 0.06 (16.5 IgA–IgG) Rumen total IgG–IgA: 0.21 (4.87 IgA–IgG)	[66]
Blood IgA	42 days after primary	3.3 (log10 units/mL) ^1^	56 days after primary			
Saliva IgG	21 days after primary	3.2 (log10 units/mL) ^1^	21 days after primary			
Saliva IgA	21 days after primary for Montanide ISA61 plus MPL^2^ 42 days after primary for Montanide ISA61	2.9 (log10 units/mL) 3.0 (log10 units/mL) ^1^	21 days after primary for Montanide ISA61 plus MPL^2^ 42 days after primary for Montanide ISA61			
Rumen IgG	21 days after primary	1.5 (log10 units/mL) ^1^	56 days after primary			
Rumen IgA	42 days after primary only for Montanide ISA61	2.9 (log10 units/mL) ^1^	42 days after primary for Montanide ISA61 plus MPL ^2^			
Blood IgG	Only one measure after vaccination	320,000.00	63 days after primary	Primary vaccination with booster at 35 and 45 days after primary with the protein rEhaF from *M. ruminantium* M1	Blood–saliva IgG: 714 Blood–rumen IgG: 60,038 Saliva–rumen IgG: 84	[86]
Saliva IgG		448.00				
Rumen IgG		5.33				
Blood IgG	21 days after primary	35% of total IgG ^1^	42 days after primary	Primary vaccination with booster at 21 days after primary, and Montanide ISA61 as adjuvant	Blood–saliva total IgG: 478 Blood–rumen total IgG: 99,655 Saliva–rumen total IgG: 209 Blood–saliva total IgA: 0.55 Blood–rumen total IgA: 65 Saliva–rumen total IgA: 117 Blood total IgG–IgA: 131 Saliva total IgG–IgA: 0.15 (6.57 IgA–IgG) Rumen total IgG–IgA: 0.09 (11.7 IgA–IgG)	[85]
Saliva IgG		42% of total IgG ^1^				

^1^ Approximate values from article figures, ^2^ Monophosphoryl lipid A, ^3^ Calculated from real and extrapolated results. rGT2 (recombinant glycosyl transferase protein).

**Table 3:** Effect of research into vaccinating ruminants on methane production.

Methane Production	Compared Groups	Conditions	References
12.8/14.8% ^1^ methane reduction in vitro	Sheep vaccinated with methanogen mix vs. prevaccinated/vaccinated with adjuvant or PBS	Primary vaccination with booster 28 days after primary Methane production from rumen liquor incubated for 24 h	[78]
26.26% ^1^ methane reduction in vitro	Sheep vaccinated with methanogens mix vs. adjuvant and PBS	Primary vaccination with booster 28 days after primary Methane production from rumen liquor incubated for 24 h, corrected for dry-matter intake	
Unsuccessful in vivo	Sheep vaccinated with mixes of three or seven methanogens vs. adjuvant and PBS	Primary vaccination Methane production on day 56 or 70 after primary	[79]
12.8% methane reduction in vivo 7.7% methane reduction in vivo, corrected for dry-matter intake	Sheep vaccinated with mix of three methanogens vs. adjuvant and PBS	Primary vaccination with revaccination 153 days after primary Methane production 180–195 days after primary	
Unsuccessful in vivo	Sheep vaccinated with mix of seven methanogens vs. adjuvant and PBS		
Unsuccessful in vivo	Sheep vaccinated with three methanogens vs. adjuvant	Primary vaccination with booster 42 days after primary Methane production 28 days after vaccination	[80]
	Sheep vaccinated with three methanogens plus additional methanogens vs. adjuvant		
Unsuccessful in vitro	Three semipurified IgY from hens vaccinated with three methanogens vs. semipurified IgY from prevaccinated hens	Primary vaccination with booster on Days 21, 42, 84, and 133 Methane production from rumen liquor incubated for 24 h	[82]
20% methane increase with anti-*Methanobrevibacter ruminantium* IgY 15% methane increase with anti-*M. smithii* IgY corrected for dry-matter disappearance	Three freeze-dried egg powders from hens vaccinated with three methanogens vs. freeze-dried egg powder from prevaccinated hens	Primary vaccination with booster on Days 21 and 42 Methane production from rumen liquor incubated for 3 h	
34% methane reduction with anti-*M. smithii* IgY 52% methane reduction with anti- *Methanosphaera stadtmanae* IgY 66% methane reduction with their combination, corrected for dry-matter disappearance		Primary vaccination with booster on Days 21 and 42 Methane production from rumen liquor incubated for 12 h	
Unsuccessful		Primary vaccination with booster on Days 21 and 42 Methane production from rumen liquor incubated for 24 h	
49–69% reduction, corrected for dry-matter disappearance	Freeze-dried egg powder from pre-vaccinated hens vs. without egg powder addition	Primary vaccination with booster on Days 21 and 42 Methane production from rumen liquor incubated for 3, 12, and 24 h	
Unsuccessful in vivo	Sheep vaccinated with five methanogens vs. adjuvant and PBS	Primary vaccination with booster on Day 28 and revaccination at Day 103 Methane production between 34 and 42 days after first booster and between 24 and 33 days after revaccination	[81]
29% ^1^ methane reduction in vitro	Sera from sheep vaccinated with *M. ruminantium* M1 whole cells vs. prevaccinated sheep sera	Primary vaccination with booster on Day 21 Methane production from methanogen culture incubated for 22 h with sera	[83]
40% ^1^ methane reduction in vitro	Sera from sheep vaccinated with *M. ruminantium* M1 cytoplasmic fraction vs. pre-vaccinated sheep sera		
Unsuccessful in vitro	Sera from sheep vaccinated with *M. ruminantium* M1 wall fraction vs. prevaccinated sheep sera		
Unsuccessful in vitro	Sera from sheep vaccinated with *M. ruminantium* M1 wall fraction with trypsin vs. prevaccinated sheep sera		
40%^1^ methane reduction in vitro	Sera from sheep vaccinated with derived-protein *M. ruminantium* M1 wall fraction vs. prevaccinated sheep sera		
Unsuccessful in vivo	Goat vaccinated with protein rEhaF from *M. ruminantium* M1 vs. animal vaccinated with elution buffer plus adjuvant	Primary vaccination with boosters on Day 35 and 45 after primary. Methane measured 60–62 days after primary	[86]

^1^ Approximate values from article figures.
